# Development of a Novel Assay Based on Plant-Produced Infectious Bursal Disease Virus VP3 for the Differentiation of Infected From Vaccinated Animals

**DOI:** 10.3389/fpls.2021.786871

**Published:** 2021-12-07

**Authors:** Alessio Bortolami, Marcello Donini, Carla Marusic, Chiara Lico, Charifa Drissi Touzani, Federica Gobbo, Elisa Mazzacan, Andrea Fortin, Valentina Maria Panzarin, Francesco Bonfante, Selene Baschieri, Calogero Terregino

**Affiliations:** ^1^Department of Comparative Biomedical Sciences, Istituto Zooprofilattico Sperimentale delle Venezie (IZSVe), Legnaro, Italy; ^2^Laboratory of Biotechnology, ENEA Casaccia Research Center, Rome, Italy; ^3^Avian Pathology Unit, Pathology and Veterinary Public Health Department, Agronomic and Veterinary Institute Hassan II, Rabat, Morocco

**Keywords:** infectious bursal disease virus, VP3, plant molecular farming, agroinfiltration, diagnostic ELISA, differentiating infected from vaccinated animals (DIVA)

## Abstract

Infectious bursal disease virus is the causative agent of Gumboro disease, a severe infection that affects young chickens and is associated with lymphoid depletion in the bursa of Fabricius. Traditional containment strategies are based either on inactivated or live-attenuated vaccines. These approaches have several limitations such as residual virulence or low efficacy in the presence of maternally derived antibodies (MDA) but, most importantly, the impossibility to detect the occurrence of natural infections in vaccinated flocks. Therefore, the development of novel vaccination strategies allowing the differentiation of infected from vaccinated animals (DIVA) is a priority. Recently, commercial vectored and experimental subunit vaccines based on VP2 have been proved effective in protecting from clinical disease and posed the basis for the development of novel DIVA strategies. In this study, an engineered version of the VP3 protein of IBDV (His-VP3) was produced in plants, successfully purified from *Nicotiana benthamiana* leaves, and used to develop an enzyme-linked immunosorbent assay (ELISA) for the detection of anti-VP3 antibodies. The His-VP3 ELISA was validated with a panel of 180 reference sera and demonstrated to have 100% sensitivity (95% CI: 94.7–100.0) and 94.17% specificity (95% CI: 88.4–97.6). To evaluate the application of His-VP3 ELISA as a DIVA test, the novel assay was used to monitor, in combination with a commercial kit, detecting anti-VP2 antibodies, the immune response of chickens previously immunized with an inactivated IBDV vaccine, a recombinant Turkey herpes virus carrying the VP2 of IBDV (HVT-ND-IBD) or with plant-produced VP2 particles. The combined tests correctly identified the immune status of the vaccinated specific pathogen free white-leghorn chickens. Moreover, the His-VP3 ELISA correctly detected MDA against VP3 in commercial broiler chicks and showed that antibody titers fade with time, consistent with the natural decrease of maternally derived immunity. Finally, the novel assay, in combination with a VP2-specific ELISA, demonstrated its potential application as a DIVA test in chickens inoculated with VP2-based vaccines, being able to detect the seroconversion after challenge with a very virulent IBDV strain.

## Introduction

Infectious bursal disease virus is the etiological agent of an acute, highly contagious viral disease that affects young chickens worldwide. IBDV is a double-stranded RNA (dsRNA) birnavirus, a member of the genus *Avibirnavirus*, of which two different serotypes (serotypes 1 and 2) are currently recognized (Eterradossi and Saif, 2019). Serotype 1 viruses can be further differentiated in classical and variant strains on the basis of their antigenic type (Mahgoub, 2012). Both classical and variant serotype 1 IBD viruses are pathogenic in chickens, causing mortality and immunosuppression, while serotype 2 viruses infecting both chickens and turkeys are nonpathogenic (Sharma et al., 2000). Severe clinical disease, bursal atrophy, and mortality are observed if infection occurs between 3 and 6 weeks of age, while if infection occurs in the first 3 weeks of life, immunosuppression is usually the only consequence (Kegne and Chanie, 2014). The immunosuppression induced by field or live attenuated viruses in growing chickens increases the vulnerability to various types of infections and reduces the response to vaccination against other pathogens (Mazariegos et al., 1990; Thangavelu et al., 1998). The worldwide distribution, the severe consequences of the infection, and the challenges in developing effective control strategies make IBD one of the most important diseases affecting commercial chickens (Van Den Berg, 2000; Eterradossi and Saif, 2019).

IBDV genome consists of two linear segments (A and B) for a total of 6 kb in length. The genome encodes for five proteins designated VP1, VP2, VP3, VP4, and VP5, of which VP1, VP2, and VP3 are structural. VP2 and VP3 are the most abundant proteins in mature virions and account for 51 and 40% of the viral proteins, respectively (Dobos et al., 1979). VP2 is the main immunogenic protein of IBDV. It induces virus-neutralizing antibodies and is responsible for antigenic variation, tissue-culture adaptation, and viral virulence (Brandt et al., 2001). VP3, the other major structural protein, is more conserved among strains and is immunogenic in chickens. However, the immunity induced by VP3 is not protective against clinical disease (Palka et al., 2021).

Traditional IBD control strategies are based on producing chicks with high levels of maternally derived antibodies (MDA) by hyperimmunizing dams with IBDV-inactivated vaccines. But, although MDA can provide variable levels of protection during the first few weeks of life, to maintain protection, vaccination is needed (Bublot et al., 2007). Current prevention strategies against IBD in young chickens are mainly based on modified live vaccines (MLV) or on inactivated, immune complex (Icx) and vectored vaccines (Müller et al., 2012). MLV are classified based on their degree of attenuation as “mild,” “intermediate,” and “hot.” “Mild” MLV has limited effect on chicken bursae, and their efficacy in the presence of high levels of IBDV MDA and against very virulent IBDV (vvIBDV) strains is poor. “Intermediate” and “hot” MLV can induce better protection against more virulent strains and are less affected by MDA but can induce moderate to severe bursal lesions (Kumar et al., 2000; Rautenschlein et al., 2005). Inactivated whole-IBDV-based vaccines are mostly formulated as water-in-oil emulsions and are generally used in prime-boost regimens in layers after priming with MLV. In an attempt to reduce the residual virulence of live vaccines, IBDV-Icx vaccines have been developed, combining MLV and IBDV-specific hyperimmune chicken serum. These vaccines allow *in ovo* vaccination and, in experimental challenges, have demonstrated similar to greater efficacy compared to MLV (Giambrone et al., 2001). More recently, efforts in IBD vaccine development have focused the attention on providing immunity only toward the viral capsid protein VP2, the major protective IBDV antigen (Letzel et al., 2007). The VP2 protein, encoded by genomic segment A and derived from a large precursor protein (VP0) by a series of proteolytic processes, hosts conformation-dependent immune determinants that control antibody-dependant neutralization and protection (Schnitzler et al., 1993; Zanetti et al., 2012). Live recombinant viruses have been engineered to express the VP2 protein and used to formulate vaccines that elicit protective immune responses against IBDV. Among these formulations, those based on the Turkey herpesvirus (HVT) have been licensed in many countries for *in ovo* or subcutaneous delivery in 1-day-old chickens (Bublot et al., 2007; Le Gros et al., 2009). More cost-effective experimental VP2-based subunit vaccines have also been developed using different expression systems, such as *Escherichia coli* (Rong et al., 2007), yeasts (Cai et al., 2013; Taghavian et al., 2013), insect cells (Hu et al., 1999; Liu et al., 2005), and plant species (Wu et al., 2004; Lucero et al., 2019; Marusic et al., 2021). Recently, a prototype vaccine based on supramolecular structures resulting from the self-assembly of the VP2 has been produced in *Nicotiana benthamiana* plants and was able to confer protection to challenge with a vvIBDV strain and to prevent the onset of major histo-morphological alterations of the bursa of Fabricius (Marusic et al., 2021). From a general point of view, the adoption of the suggested plant “biofactory” approach in the veterinary field has the potential to result in: i) ease and rapidity of production scale-up at low costs; ii) improvement of the immunogenic properties of the antigens obtained by self-assembly in multimeric structures; iii) development of low-cost and ready-to-use DIVA diagnostic tools for surveillance programs (Rage et al., 2020). Both viral vectored and VP2-based vaccines have demonstrated good efficacy in protecting chickens from clinical IBD in experimental and field trials (Perozo et al., 2009; Müller et al., 2012; Rage et al., 2019).

With the aim to develop a DIVA strategy for IBD in chickens immunized with commercial and experimental new-generation VP2-based vaccines, we produced in *N. benthamiana* plants the recombinant VP3 protein and devised an indirect enzyme-linked immuno-sorbent assay (ELISA) that offers the opportunity to better control one of the most important diseases for the poultry industry.

## Materials and Methods

### Plant-Expression Constructs and Agroinfiltration of *N. benthamiana* Plants

**Figure 1:**
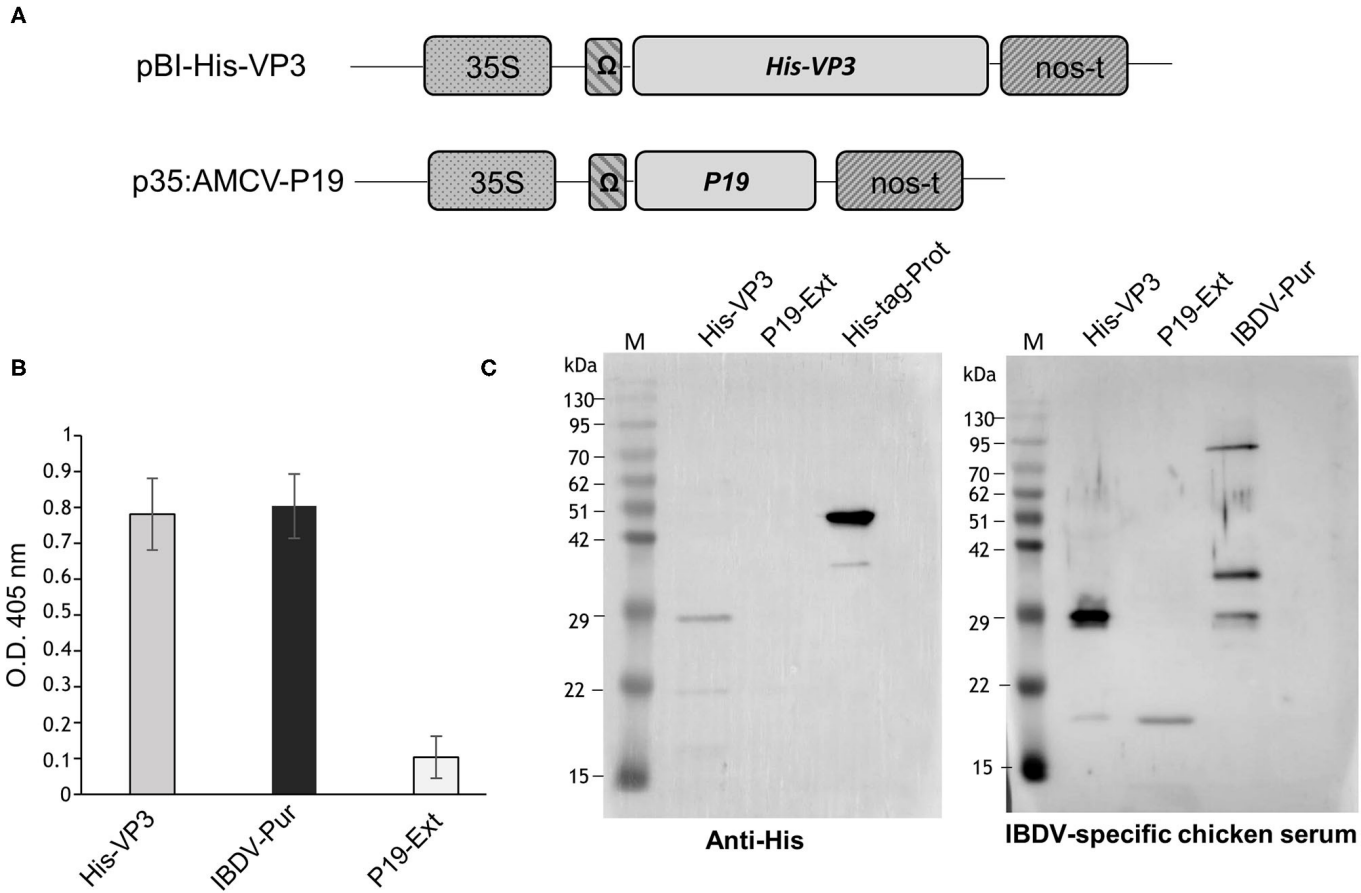
Expression of His-VP3 in *N. benthamiana* plants. **(A)** Schematic representation of pBI-His-VP3 and p35:AMCV-P19 plant expression vectors. 35S: CaMV 35S promoter; Ω: TMV translational enhancer sequence; *His-VP3*: sequence encoding the IBDV VP3 protein N-terminally fused to a 6X histidine tag; *P19*: sequence encoding the AMCV p19 gene-silencing suppressor; nos-t: Nopaline synthase terminator from *A. tumefaciens*. **(B)** ELISA assay of TSP extracted from leaves agroinfiltrated with pBI-His-VP3 and p35:AMCV-P19 and collected at 7 DPI. Fifty μg of TSP were distributed in triplicate into the wells, and His-VP3 was detected with a chicken anti-IBDV serum. The reported values are the mean of three independent experiments, and error bars represent the SD of the means. IBDV-Pur: wells coated with purified inactivated IBDV (30 ng/well); P19 Ext: wells coated with the extract from leaves agroinfiltrated only with p35:AMCV-P19, used as the negative control. **(C)** Western blot analysis of His-VP3 expression in leaf extracts. Ten μg of TSP was separated by 12% SDS-PAGE. Left panel: His-VP3 detection using an anti-His antibody. M: A protein molecular weight marker; His-VP3: His-VP3 plant extract; P19 Ext: extracts from leaves agroinfiltrated only with p35:AMCV-P19, used as negative control; His-tag-Prot: unrelated His-tagged protein used as the positive control. Right panel: His-VP3 detection using the chicken anti-IBDV serum. M: A protein molecular weight marker; His-VP3: His-VP3 plant extract; P19 Ext: extracts from leaves agroinfiltrated only with p35:AMCV-P19, used as negative control; IBDV-Pur: purified inactivated IBDV used as the positive control.

The VP3 sequence was derived from an IBDV strain (IZSVE L1/08) of the IZSVe collection. The synthetically constructed *His-VP3* gene encoding a hexa-histidine tag (His) fused to the N-terminus of the entire sequence of the VP3 protein (GenBank accession No. OK257849) has been optimized according to the codon bias of *N. benthamiana* (http://www.kazusa.or.jp/codon) using the GENEius software (Eurofins Genomics, Ebersberg, Germany). The synthetic sequence (Eurofins Genomics, Ebersberg, Germany) has been excised from pEX by digestion with *Bam*HI and *Xma*I restriction enzymes and inserted into similarly digested pGEM-NOS plasmid (Marusic et al., 2007) to be then transferred, together with the *nopaline synthase* gene terminator (NOSter) of *Agrobacterium tumefaciens*, into the binary vector pBI-Ω using *Bam*HI/*Eco*RI restriction sites, yielding the pBI-His-VP3 construct (Figure 1A). In this vector, gene expression is under the control of the constitutive Cauliflower Mosaic Virus 35S promoter (35SCaMV), the Ω translational enhancer sequence from Tobacco Mosaic virus, and the NOSter sequence.

The p35:AMCV-P19 construct (Figure 1A), harboring the *p19* gene encoding the Artichoke Mottled Crinkle virus (AMCV) P19 silencing suppressor protein, was previously described (Lombardi et al., 2009). The pBI-His-VP3 and the p35: AMCV-P19 vectors were separately introduced in *A. tumefaciens* LBA4404 (Thermo Fisher Scientific, Rockford, IL, USA) and the two transformed strains were separately grown in the Luria Bertani medium overnight at 28°C. The cells were centrifuged at 4,000 x g and pellets resuspended in the infiltration buffer (10 mM MES, 10 mM MgCl_2_, pH 5.8) to reach the optical density at a wavelength of 600 nm (O.D._600_) of 0.6 for each clone and then mixed in a 1:1 ratio before agroinfiltration. Transient expression of the recombinant His-VP3 was obtained in *N. benthamiana* plants grown at the 6–7 leaf stage in a greenhouse at 24°C under controlled conditions (16/8-h light/dark cycle). The aerial part of the plants was immersed in the bacterial suspension and infiltration performed in a vacuum chamber, applying a pressure of ~10 mmHg. Leaf samples were harvested at 7 days post infiltration (DPI), frozen in liquid N_2_, and stored at −80°C. Some plants were infiltrated only with *A. tumefaciens*, harboring p35: AMCV-P19 as negative control (C-).

### Recombinant Protein Extraction and Purification

The plant tissues (200 mg) were ground in liquid N_2_ and homogenized in 400 μl of phosphate-buffered saline (PBS) pH 7.2, containing a protease inhibitor cocktail (cOmplete^TM^; Roche, Mannheim, Germany). Plant extracts were clarified by centrifugation at 20,000 x g for 20 min, and the total soluble protein (TSP) content was determined by Bradford colorimetric assay, following the manufacturer's protocol (Bio-Rad Protein Assay, Hercules, CA, USA).

For His-VP3 purification, leaves have been ground in liquid N_2_, mixed 1:2 with Buffer 1 (300 mM KCl, 50 mM KH_2_PO_4_, and 5 mM Imidazole), and homogenized using Ultraturrax T 25 (IKA, Staufen, Germany). After Miracloth (Sigma Aldrich, Saint Louis, MO, USA) paper filtration and centrifugation at 22,000 × g, 20 min at 4°C, the supernatant has been further centrifuged two times at 30,000 x g for 15 min. The clarified extract has been filtered through a 0.45 μm syringe filter (Millipore, Bedford, MA, USA) and then passed through an Immobilized Metal Affinity Chromatography (IMAC) column (Bio-Scale Mini Profinity IMAC Cartridge, 1 ml, Bio-Rad, Hercules, CA, USA) according to the instructions of the manufacturer. Elution (0.5 ml fractions) has been performed using Native Elution Buffer (300 mM KCl, 50 mM KH_2_PO_4_, and 250 mM Imidazole). VP3-containing fractions have been dialyzed and concentrated in PBS using Vivaspin 2 columns (5000 MWCO HY, Sartorius, Gottinga, Germany). The protein concentration was determined by measuring absorbance at 280 nm.

### Recombinant Protein Analysis

For the detection of His-VP3 by ELISA, 50 μg TSP of each clarified extract or 30 ng of purified inactivated IBDV was distributed in 100 μl in triplicate into the wells of Nunc-Immuno Maxisorp plates (NUNC, Roskilde, Denmark) and incubated at 4°C overnight. After washing three times with PBS containing 0.1% Tween20 (PBS-T) and two times with PBS, the plates were incubated with 2% (w/v) milk in PBS (PBS 2% M) at 37°C, for 2 h. Plates were washed again and a chicken anti-IBDV serum (Rage et al., 2019) was diluted 1:200 in PBS 2% M and then added and incubated at 37°C for 2 h. For the purified VP3 analysis serial dilutions (from 1:200 to 1:1,000), PBS 2% M of the chicken anti-IBDV serum or sera from healthy or NDV-infected chickens as controls was used. The detection was performed incubating the wells for 1 h at 37°C with a goat anti-IgY Horse Radish Peroxidase (HRP)-conjugated antibody (A9046, Sigma-Aldrich, Saint-Louis, MO, USA) diluted 1:2,000 in PBS 2% M and then revealing the enzymatic activity, adding to the wells 2,2-azino-di-3-ethylbenz- thiazoline sulphonate (KPL, Milford, MA, USA). The plates were read at 405 nm by a microtiter plate reader (TECAN-Sunrise, Groedig, Austria). Inactivated IBDV (Rage et al., 2019) was used as a positive control (IBDV-Pur), while the extract of plants infiltrated only with p35:AMCV-P19 as negative control (P19-Ext).

To perform Western blot analysis, extracts from His-VP3 infiltrated plants containing 10 μg of TSP or concentrated pools of the fractions obtained by IMAC (4 μl) were separated by 12% SDS-PAGE under reducing conditions by adding 3% β-mercaptoethanol into the sample loading buffer (50-mM Tris HCl pH 6.8, 2% w/v SDS, 10% glycerol, 0.1% w/v bromophenol blue) in parallel with P19 plants extract (10 μg TSP; P19 Ext, negative control), 50 ng of an unrelated His-tagged protein (His-tag-Prot) or 30 ng of purified inactivated IBDV (IBDV-Pur) (positive controls). Proteins were electro-transferred to polyvinylidene fluoride (PVDF) membranes (Millipore, Bedford, MA, USA) using a Semi-Dry Transfer Unit (Hoefer TE70; GE Healthcare, Freiburg, Germany), which were then blocked with PBS 4% M 2 h at room temperature. The membranes were incubated overnight at 4°C with a chicken anti-IBDV serum (Rage et al., 2019) or with a mouse anti-His tag monoclonal antibody (Roche, Mannheim, Germany), both diluted 1:200 in PBS 2% M and then with the goat anti-IgY Horse Radish Peroxidase (HRP)-conjugated antibody (A9046, Sigma-Aldrich, Saint-Louis, MO, USA) diluted 1:2,000 in PBS 2% M 1 h at 37°C or the anti-mouse HRP-conjugated antibody (KPL, Milford, MA, USA) diluted 1:5,000 1 h at room temperature, respectively. Between each incubation, the membranes were washed as described for ELISA. Proteins were detected by enhanced chemiluminescence (ECL Plus; GE Healthcare, Uppsala, Sweden) using an ImageQuant^TM^ LAS 500 system (GE Healthcare, Uppsala, Sweden). Purified VP3 fractions were also separated by 12% SDS-PAGE under reducing conditions, and the gel was stained with Coomassie R250 (Sigma Aldrich, Saint-Louis, MO, USA).

### Sera Used in Optimization and Validation of the Novel Assay

To optimize and assess the performances of the novel His-VP3-based ELISA, a wide range of sera was obtained from White Leghorn Specific Pathogen Free (WL SPF) and broiler chickens in previous immunization experiments or from virologically confirmed IBD outbreaks were used (Table 1). Immune status was confirmed with the commercial VP2-based ELISA (indirect ELISA kit ID Screen® IBD VP2; ID Vet, Grabels, France). To examine the specificity of the His-VP3 ELISA, positive serum samples from the IZSVe serum archive against avian influenza virus (subtypes H5N1, H7N3, and H9N2), avian reovirus (ARV), avian leukosis virus (ALV, subgroup J), chicken anemia virus (CAV), infectious bronchitis virus (IBV, M41), infectious laryngotracheitis virus (ILTV), Newcastle Disease virus (NDV, strain Ulster), and pigeon paramyxovirus serotype 1 (PPMV-1) were tested.

**Table 1:** Details of the source of the sera used for His-VP3 ELISA optimization and performances evaluation.

**Sample category**	**IBDV-immune status**	**Number of samples**	**Source (reference)**
White Leghorn (WL) SPF Chicken	Naïve (SPF-Naïve)	102	IZSVe experimental serum archive
WL SPF Chicken	vvIBDV experimentally infected – recovered (SPF-IBDV-Recovered)	10	IZSVe experimental serum archive
WL SPF Chicken	Vaccinated with inactivated D78 strain (SPF-Inactivated IBDV Vaccinated)	18	Previous study (Marusic et al., 2021)
WL SPF Chicken	Plant-produced VP2 immunized (VP2-VLP vaccinated)	18	Previous study (Marusic et al., 2021)
Commercial Broilers	Naturally Infected	40	Field sera – IZSVe serum archive

*IZSVe, Istituto Zooprofilattico Sperimentale delle Venezie; SPF, specific pathogen free*.

### Experimental Immunization With HVT-IBD-ND in WL SPF and Commercial Broilers

Twenty-two WLSPF and 12 broiler chickens (Ross X Cobb) were allocated in Biosafety Level 3 (BSL3) poultry isolators (Montair, The Netherlands) immediately the following hatch. Twelve SPF and 12 broiler chickens were immunized at Day 1 post-hatch with a commercial double HVT vector vaccine (HVT-ND-IBD), following the instructions of the manufacturer. The remaining 10 SPF chickens were sham vaccinated with PBS and kept as non-immunized controls. Blood samples were taken from all the birds at 14, 21, 28 days of age (before the challenge) and Day 14 postinfection (p.i.). All the birds were challenged with a field vvIBDV strain (L1/08) at 10^5^ BID_50_ at 28 days of age by the oronasal route. On Days 4 and 7 p.i., cloacal swabs (FL Medical, Torreglia, Italy) were collected from all surviving birds to evaluate cloacal viral shedding by real-time reverse transcription polymerase chain reaction (rRT-PCR). Animal experiment procedures were conducted in strict accordance with the Decree of the Italian Ministry of Health n. 26 of 4 March 2014 on the protection of animals used for scientific purposes, implementing Directive 2010/63/EU, and approved by the Ethics Committee of IZSVe (Authorization No: n° 709/2020-PR). The animals were kept within IZSVe BSL3 animal facilities with feed and water *ad libitum*.

### His-VP3 ELISA

Dilutions of purified His-VP3 antigen and a goat anti-chicken serum conjugated to HRP (Novex, Life technologies, UK) were optimized in a chessboard format with positive and negative sera (Supplementary Figure 1). In the following experiments, 30 ng of purified VP3 in a 100 μl coating buffer (0.5 M carbonate/bicarbonate, pH 9.6) was used and distributed into the wells of Nunc-immuno MaxiSorp™ plates (Thermo Fisher Scientific, Waltham, MA, USA) and incubated overnight at 4°C. To remove unbound proteins, the plates were washed three times with PBS-T. Coated wells were then incubated for 1 h at room temperature with a blocking solution (2% BSA in PBS) to reduce non-specific binding, and with 100 μl of chicken sera diluted 1:200 in blocking buffer PBS, 0.05% Tween 20, 1% BSA, for 2 h at room temperature. After washings with PBS-T three times aimed to remove unbound antibodies, the wells were incubated with 100 μl of a conjugated rabbit anti-chicken serum (1:20,000) for 1 h at room temperature. After five washes, wells were incubated for 7 min at room temperature with Tetramethylbenzidine (TMB) substrate (Sera Care, Milford, MA, USA), and coloring was stopped by the addition of the stop solution (2N H_2_SO_4_). Optical density at 450 nm (O.D._450_) was read with a Tecan Sunrise^TM^ spectrophotometer microplate reader (Tecan, Groedig, Austria).

Evaluation of the assay reproducibility within and between runs was performed with nine serum samples, three strong positive, three moderately positive, and three negative sera. For intra-assay (within-plate) reproducibility, three replicates of each serum sample were analyzed within the same plate, and the experiment was performed three times independently. For inter-assay (between-run) reproducibility, three replicates of each serum sample were run on different plates. This test was performed two times using plates coated at different times. The mean O.D._450_ values, standard deviation (SD), and coefficient of variation (CV) were calculated.

### VP2-Based ELISA

The commercial indirect ELISA kit ID Screen® IBD VP2 (ID Vet, Grabels, France) was used to detect specific VP2 antibodies, following the instructions of the manufacturer. O.D._450_ values were measured with a Tecan Sunrise^TM^ microplate reader (Tecan, Groedig, Austria). Positive and negative sera used were included in the kit, and positivity was set according to the instructions of the manufacturer.

### Viral Detection by rRT-PCR

Cloacal swabs were resuspended with 500 μl PBS containing antibiotics and antimycotics (1X Antibiotic-Antimycotic Solution, Sigma-Aldrich, Saint-Louis, MO, USA) (PBS-A). Nucleic acids were isolated from 300 μl of swab suspension with the QIAsymphony® DSP Virus/Pathogen Midi Kit on a QIAsymphony® SP instrument using a custom protocol provided by Qiagen (Hilden, Germany).

IBDV genome was detected by rRT-PCR targeting the VP4 gene (Peters et al., 2005) modified to a simple setup, using the QuantiTect® Multiplex RT-PCR Kit (Qiagen®, Hilden, Germany). Briefly, each reaction contained 12.5 μl of 2x QuantiTect® Multiplex RT-PCR Master Mix, 250 nM of each primer, 300 nM of a probe targeting very virulent IBDV strains (FAM – 5′-CAACGCCTATGGCGAGATTGAGAACGTGAG-3′- TAMRA),0.25 μl of QuantiTect® Multiplex RT Mix, 5 μl of template RNA and RNase-free water up to 25 μl. rRT-PCRs were run on Rotorgene 6000 (Qiagen®, Hilden, Germany) under the following cycling conditions: 50°C for 20 min and 95°C for 15 min, followed by 40 cycles at 94°C for 45 s and 60°C for 45 s. Each sample was tested in duplicate. rRT-PCR amplification data were analyzed with the Rotorgene Q series software (Qiagen®, Hilden, Germany).

### Statistical Analysis

The cut-off point and analytical sensitivity (Se) and specificity (Sp) were assessed by receiver operating characteristic (ROC) curve analysis (MedCalc Software version 17.9, 2017) using the sera obtained from previous experimental trials or the serum archive of IZSVe. True positive sera belonged to animals naturally infected or vaccinated from previous experiments or commercial farms. Negative sera came from SPF chickens used in past experiments in our laboratory. The Cohen kappa (κ) coefficient of agreement between the His-VP3 and the VP2 ELISA was calculated with Prism Prism v.9.1.2 (GraphPad, San Diego, CA, USA).

## Results

### Cloning and Expression of His-VP3 in N. Benthamiana Plants

The *His-VP3* gene encoding the VP3 protein fused at the N-terminus to His tag was cloned in the pBI-Ω plant expression vector (Figure 1A). *N. benthamiana* plants were infiltrated with a 1:1 mixed suspension of *A. tumefaciens* strains, carrying the pBI-His-VP3 or the p35:AMCV-P19 constructs (Figure 1A). As a negative control, a group of plants was infiltrated only with *A. tumefaciens*, bearing p35:AMCV-P19.

Leaves were harvested at 7 DPI, and the presence of His-VP3 in TSP was verified by ELISA using an anti-IBDV chicken serum (Figure 1B). Western blot analysis was performed to reveal not only the presence of His-VP3 but also of the His-tag needed for protein purification (Figure 1C). An anti-His antibody showed the presence in the His-VP3 sample of a major band of ~29 kDa, corresponding to the expected size of the recombinant protein. A major band of ~45 kDa was present in the His-tag-Prot sample, corresponding to the expected size of the unrelated His tagged protein used as control (Figure 1C, left panel).

Similarly, the analysis made with the chicken anti-IBDV serum revealed a major band of ~29 kDa in the His-VP3 sample (Figure 1C, right panel) corresponding to the molecular mass of the recombinant protein, while, in the IBDV-Pur lane (purified IBDV-inactivated virus), three bands were visible: one at ~29 kDa, corresponding to viral VP3; one at ~35 kDa, corresponding to VP2; and one at ~90 kDa, possibly indicating the presence of higher molecular weight protein aggregates (Figure 1C, right panel). The chicken serum recognized in both VP3 (His-VP3) and P19 (P19-Ext; negative control) agroinfiltrated leaf extracts a faint band with an estimated molecular mass of about 20 kDa.

### VP3 Purification

**Figure 2:**
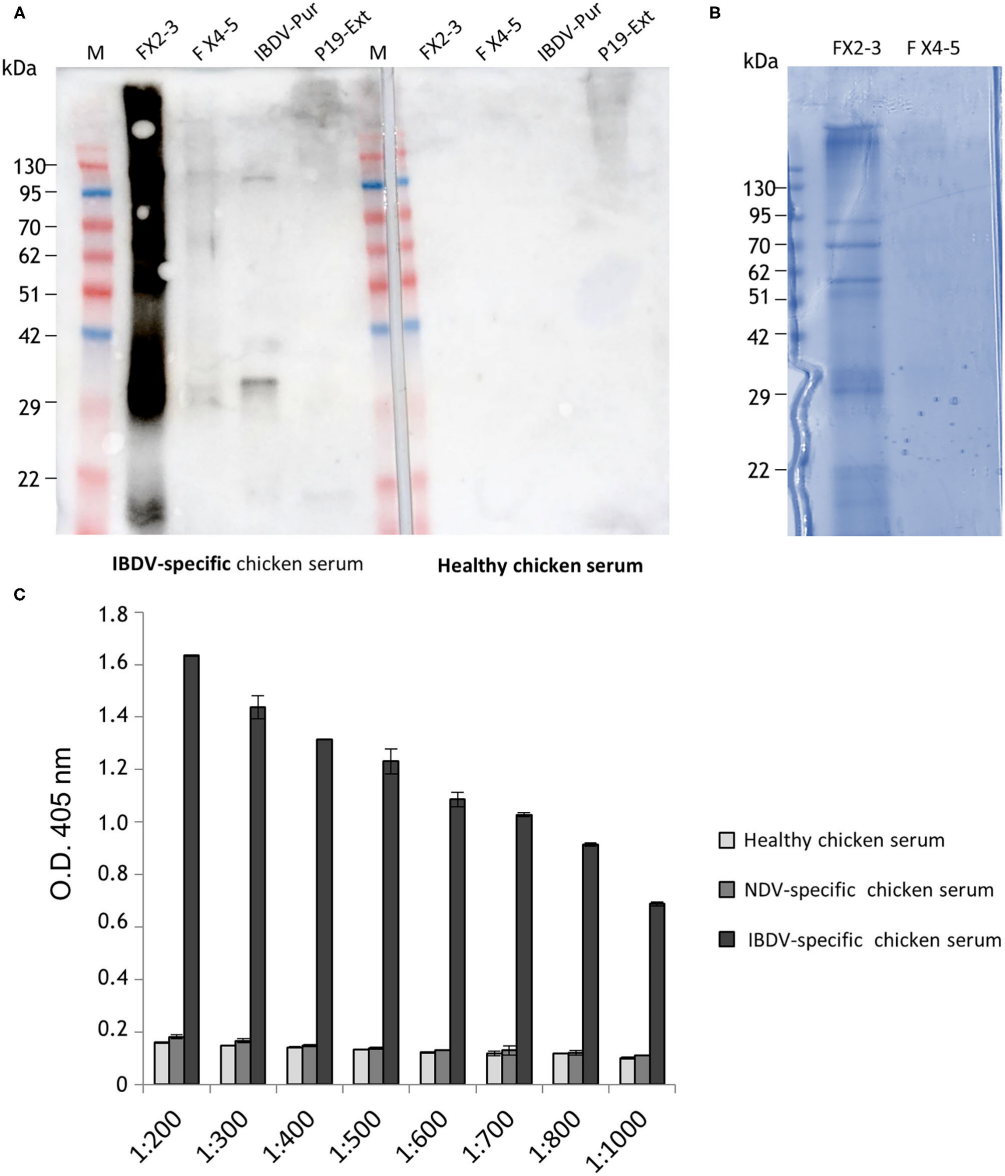
Analysis of plant-purified His-VP3. **(A)** Western blot analysis with the serum of IBDV-infected chickens of fractions obtained from IMAC and concentrated. Aliquots of the pools of Fractions 2 and 3 (FX2-3) and Fractions 4 and 5 (FX4-5) were loaded on the gel (left side of the membrane). IBDV-Pur: Purified inactivated IBDV was used as positive control; P19 Ext: extract of leaves agroinfiltrated only with P19 (negative control). The same samples were also assayed using as primary antibody the serum from healthy chickens (a negative chicken serum, right side of the membrane). M: A protein molecular weight marker. **(B)** FX2-3 and FX4-5 fractions (10 μl) separated under reducing conditions on Coomassie Blue-stained 12% SDS-PAGE. **(C)** Direct ELISA using a purified His-VP3 coated plate (30 ng per well) and serial dilutions (1:200 to 1:1,000) of an anti-IBDV chicken serum (IBDV serum) as a primary antibody. Sera from healthy chickens (negative serum) or chickens infected with an unrelated virus (Newcastle Disease Virus- NDV serum) were used as controls. The reported values are the mean of two independent experiments, and error bars represent the SD of the means.

His-VP3 was purified from plant extracts by Immobilized Metal Affinity Chromatography (IMAC). Ten fractions of 0.5 ml (FX1 to FX10) were eluted from the column and protein content was evaluated using Bradford colorimetric assay. A pool of the Fractions 2 and 3 (FX2-3), showing the highest protein content, was dialyzed and concentrated two times in PBS, reaching a final concentration of ~350 ng/μl to be then analyzed by Western blot using an IBDV-specific chicken serum or serum from healthy chickens as the negative control in parallel with a pool of less concentrated fractions (FX4-5) (Figure 2A). A strong signal was detected in the FX2-3 pool, differently from what was observed in the FX4-5 pool. In particular, a band at the expected molecular mass of ~29 kDa is visible together with higher bands, possibly corresponding to His-VP3 aggregation products. This pattern was confirmed by the Coomassie-stained SDS-PAGE analysis of FX2-3 that similarly evidenced the presence of the band at ~29 kDa and of the bands with higher molecular masses (Figure 2B). Specific recognition of His-VP3 by an anti-IBDV chicken serum used at serial dilutions was assayed by ELISA (Figure 2C). Results show a linear-decreasing signal from 1:200 to 1:1,000 dilutions. Sera from healthy chickens or chickens infected with an unrelated virus (Newcastle Disease Virus, NDV) used as negative controls did not show any specific VP3 binding.

### Accuracy and Reproducibility Evaluation of the His-VP3-Based Indirect ELISA

An indirect ELISA based on the plant-purified antigen His-VP3 was optimized using a chessboard format with positive and negative sera coating the wells with quantities of His-VP3, ranging from 0.1 to 50 ng/well (Supplementary Figure 1).

**Figure 3:**
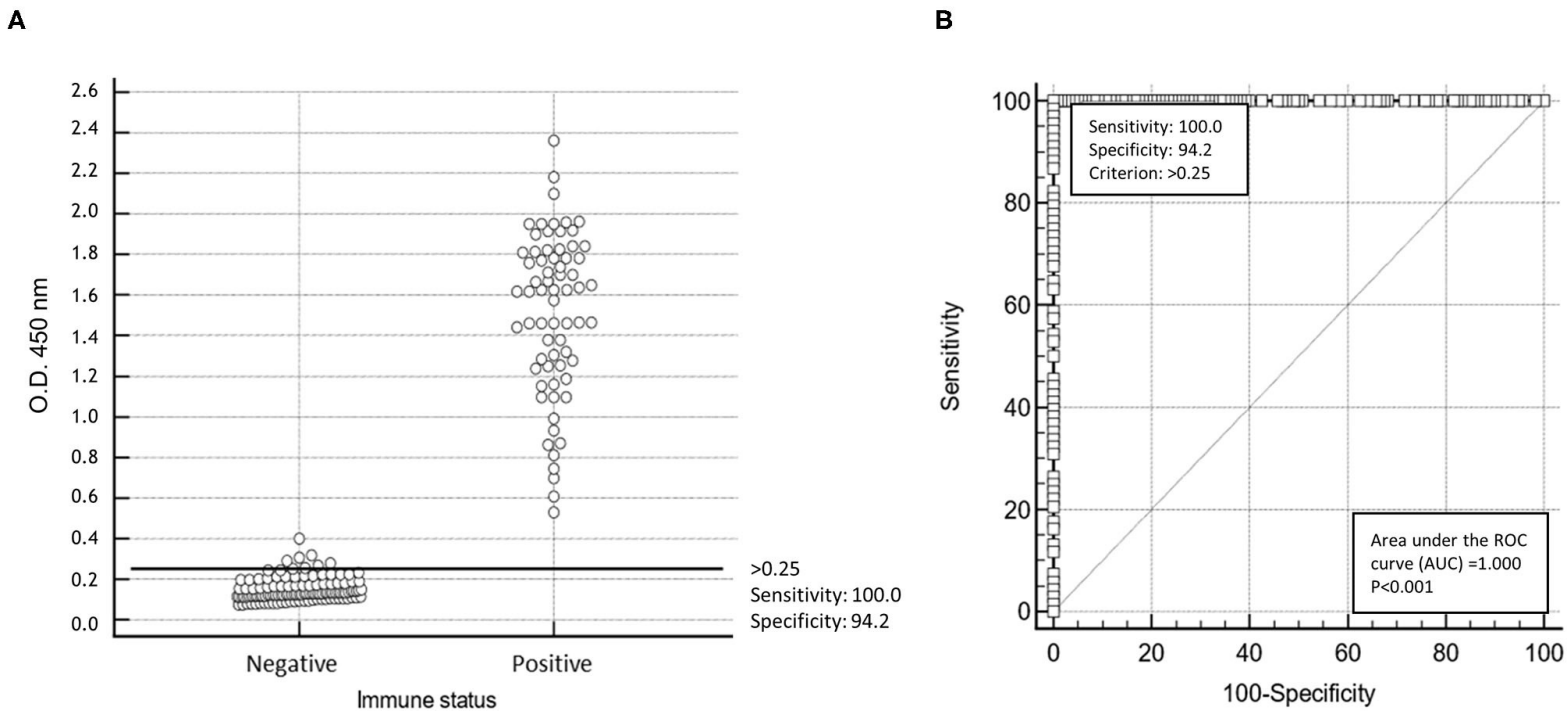
Statistical analysis of His-VP3 ELISA. **(A)** A scatter plot of 86 positive and 102 negative (0 = negative; 1 = positive) sera using cut-off point optical density (O.D._450_) > 0.25. **(B)** Receiver operating curve (ROC) analysis for determination of specificity and sensitivity performances at the established threshold (O.D._450_ = 0.250; criterion > 0.25) of His-VP3 ELISA.

The performances of this ELISA were evaluated in comparison to those of a commercial VP2 ELISA using all the sera of the collection (Table 1) at 1:200 dilution. The threshold of the His-VP3-based ELISA (O.D._450_ = 0.250) was calculated as the mean of the O.D._450_ readings of the102 naïve SPF chicken sera plus two times the SD (Figure 3A). The His-VP3-based ELISA demonstrated an analytical sensitivity of 100% (95% CI: 94.7–100) and specificity of 94.17% (95 % CI: 88.4–97.6) at the defined O.D._450_ threshold as indicated by the receiver-operating characteristic (ROC) curve analysis (Figure 3B).

Cohen's kappa value (κ) of 0.957 (CI: κ = 0.915–0.999), calculated with data obtained with the His-VP3 and the commercial VP2 ELISA, indicated an almost perfect agreement between the two assays. Specificity was further evaluated using a panel of sera raised in SPF birds against 10 pathogenic avian viruses. None of the tested sera showed reactivity against the His-VP3 (Supplementary Figure 2).

**Figure 4:**
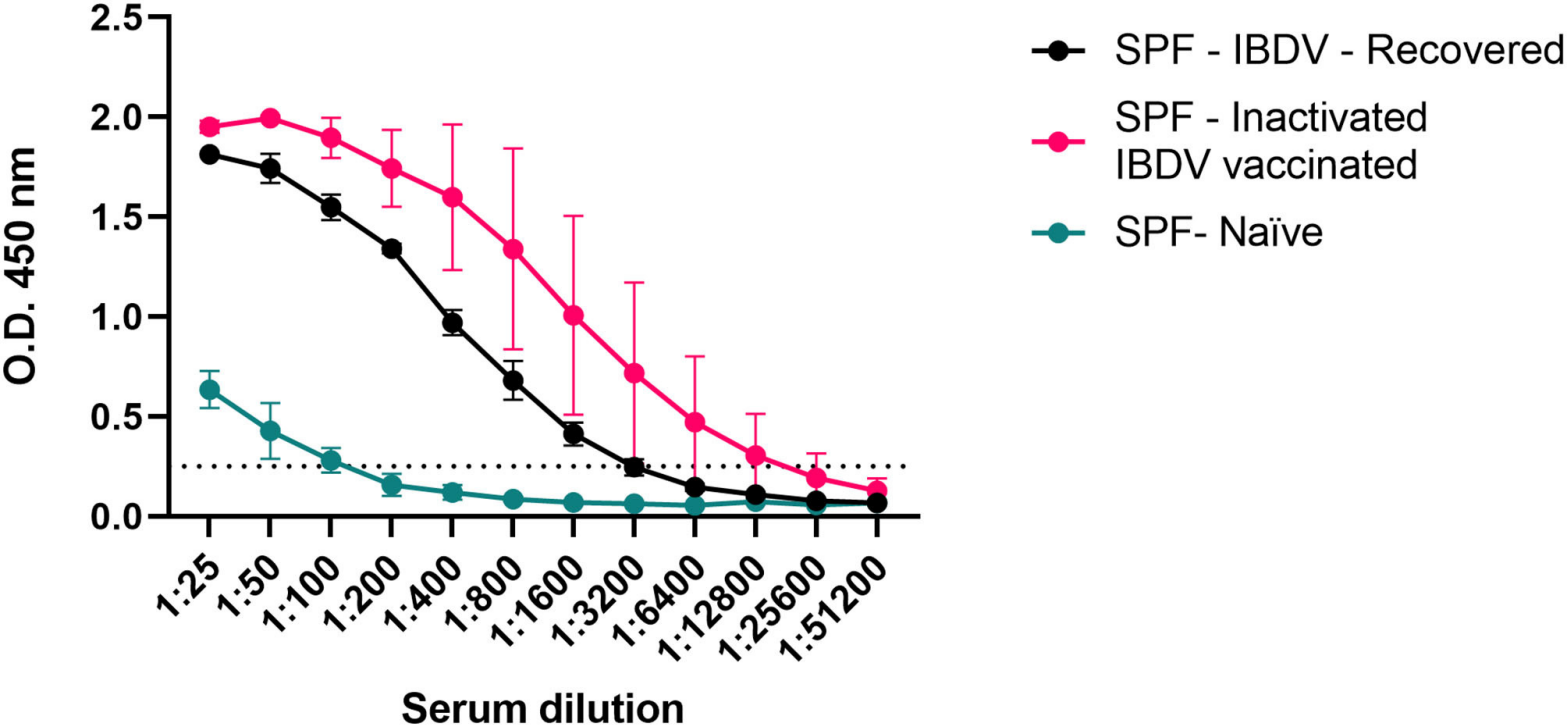
Estimation of the analytical sensitivity of the His-VP3 ELISA. Mean O.D._450_ values of three individual sera obtained from experimentally infected (SPF-IBDV-recovered; black), inactivated IBDV vaccinated (SPF-inactivated IBDV vaccinated; pink), and naïve (SPF-Naïve; green) SPF chickens. Vertical bars: standard deviation. The positive threshold for His-VP3 ELISA is indicated with a dotted line (O.D._450_ = 0.250).

To further validate the analytical sensitivity of the assay, small subsets (*n* = 3) of the sera from the experimentally infected and recovered SPF chickens (SPF-IBDV-Recovered) and from chickens vaccinated with the inactivated D78 IBDV strain (SPF-inactivated IBDV vaccinated), both expected to have developed an anti-VP3 antibody response, were serially diluted (2-fold) and tested (Figure 4). Values above the threshold were obtained up to dilution 1:1,600 for all the analyzed sera except for the sera of the naïve SPF (SPF-Naïve) chickens used as controls, confirming the high sensitivity of the assay.

To evaluate the reproducibility of the assay, three strongly positive, three weakly positive, and three negative anti-His-VP3 sera were again tested by the His-VP3-based ELISA in triplicate. The intra-assay CV ranged from 0.55 to 4.28% and the inter-assay CV from 1.25 to 4.26% (Table 2), indicating high reproducibility of the test.

**Table 2:** Reproducibility of the His-VP3-based ELISA in detecting IBDV-VP3 specific antibodies.

**Samples**		**Intra-assay variability**		**Inter-assay variability**	
		**O.D._**450**_ ± SD**	**CV (%)**	**O.D._**450**_ ± SD**	**CV (%)**
Strong positive	1	1.88 ± 0.03	1.61	1.91 ± 0.04	2.07
	2	1.92 ± 0.04	1.94	1.93 ± 0.02	1.25
	3	1.61 ± 0.03	1.72	1.64 ± 0.05	3.17
Weak positive	4	1.14 ± 0.03	2.72	1.15 ± 0.02	1.73
	5	1.28 ± 0.01	0.55	1.30 ± 0.03	2.24
	6	1.22 ± 0.02	1.72	1.22 ± 0.04	3.29
Negative	7	0.18 ± 0.00	2.25	0.18 ± 0.00	3.51
	8	0.22 ± 0.01	4.28	0.22 ± 0.01	4.03
	9	0.19 ± 0.01	3.74	0.19 ± 0.01	4.26

*Values shown are the means ± the standard deviation (SD) of three (Intra-assay variability) or six (inter-assay variability) replicates*.

### Evaluation of His-VP3-Based ELISA Efficacy to Discriminate Between Different Vaccination Strategies and IBDV Infection in SPF Chickens

To evaluate the performance of the His-VP3-based ELISA in discriminating animals infected or immunized with the inactivated vaccine from those immunized with new generation vaccines, we analyzed sera of WLSPF chickens Naïve, IBDV infected and recovered (IBDV-recovered), or vaccinated with the inactivated D78 IBDV strain (inactivated-IBDV), the registered recombinant live-vectored vaccine (HVT-ND-IBD) or the experimental plant-produced VP2-based vaccine (VP2-VLP; Marusic et al., 2021) (Table 1). In these experiments, the sera were tested in parallel also with a VP2-based ELISA.

**Figure 5:**
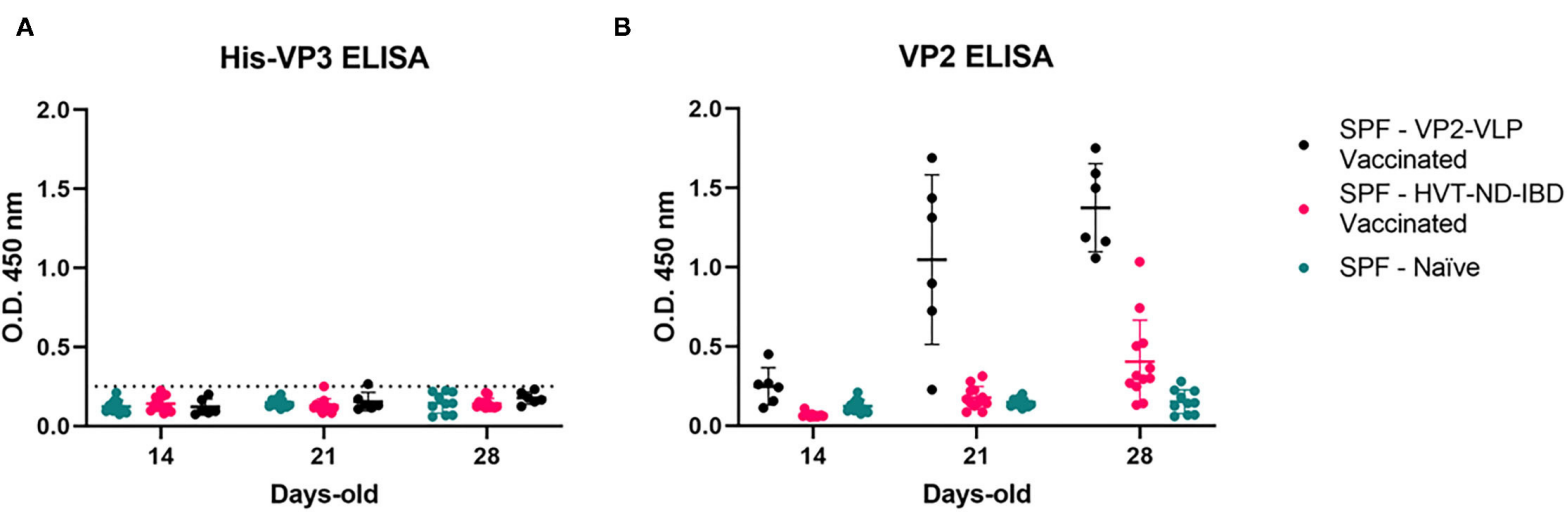
Anti-VP2 antibodies in the sera of vaccinated SPF chickens do not interfere in the His-VP3-based ELISA. Distribution of O.D._450_ values of pre-challenge SPF chickens sera collected at different time points (SPF-VP2-VLP vaccinated, black; SPF-HVT-ND-IBD vaccinated, pink; SPF-Naïve, green) analyzed with **(A)** His-VP3 or **(B)** ID Screen® IBD VP2 ELISA. Horizontal and vertical bars: mean ± standard deviation (SD). The positive threshold for His-VP3 ELISA is indicated with a dotted line (O.D._450_ = 0.250).

The animals immunized with the VP2-focused vaccines showed increasing anti-VP2 antibodies, starting from Day 21. None of the sera of these animals resulted positive in the His-VP3 ELISA, indicating that anti-VP2 antibodies do not interfere with the assay performance and could be used as a marker of vaccination when VP2-based vaccines are used (Figure 5). As expected, the sera of the Naïve chickens were negative in both assays.

**Figure 6:**
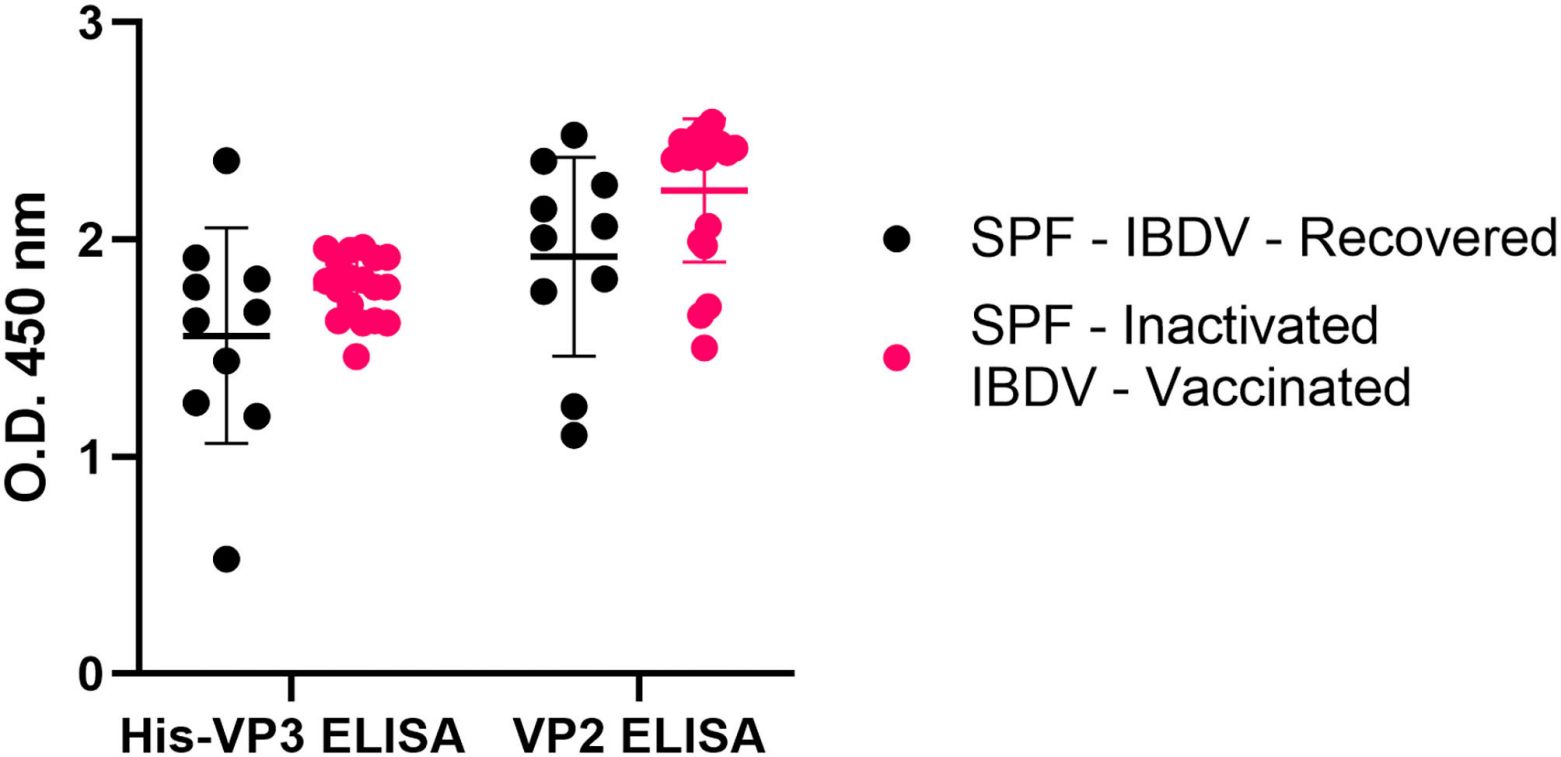
The His-VP3-based ELISA allows the identification of chicken infected or vaccinated with inactivated IBDV vaccines. Distribution of O.D._450_ values of post-challenge WL SPF chickens sera (SPF- IBDV-recovered, black; SPF-inactivated IBDV vaccinated, pink) analyzed with His-VP3 and ID Screen® IBD VP2 ELISA. Horizontal and vertical bars: mean ± standard deviation (SD).

The same analysis performed on the sera obtained from SPF chickens exposed to the whole virus (inactivated-IBDV vaccinated or IBDV-recovered) indicated that all the sera were positive for both VP2 and VP3 antibodies (Figure 6). These results demonstrated that the His-VP3 ELISA is able to detect antibodies elicited by either vaccination with inactivated virions or by IBDV infection and can be used as a marker of exposure to the whole virus. The comparable performances of the two ELISA in the analysis of these two groups of sera also indicate possible use of the novel assay as a stand-alone diagnostic test to detect infection or assess a vaccination outcome with live-attenuated or inactivated vaccines.

### Influence of MDA on His-VP3-Based ELISA Analysis of Vaccinated Broiler Chickens

Currently, control strategies against IBD are aimed at providing chicks with high MDA against the whole IBDV by vaccination of the parents with live or inactivated vaccines before the start of the laying period. As a result, chicks receive from layers both anti-VP2 and anti-VP3 antibodies. Anti-VP2 antibodies of maternal derivation, which are protective against field strains, and their decay, have been extensively studied due to their interference with IBDV vaccination. Anti-VP3 MDA is, instead, poorly studied due to the limited involvement in protection against disease. However, because, in chickens with MDA against the whole virus and vaccinated with VP2-focused vaccines, the detection of antibodies against VP3 would help to reveal the introduction of IBDV in the field, it is of outmost importance to study the kinetics of anti-VP3 antibodies in these animals, identifying the earliest time point at which this MDA is no longer detectable.

**Figure 7:**
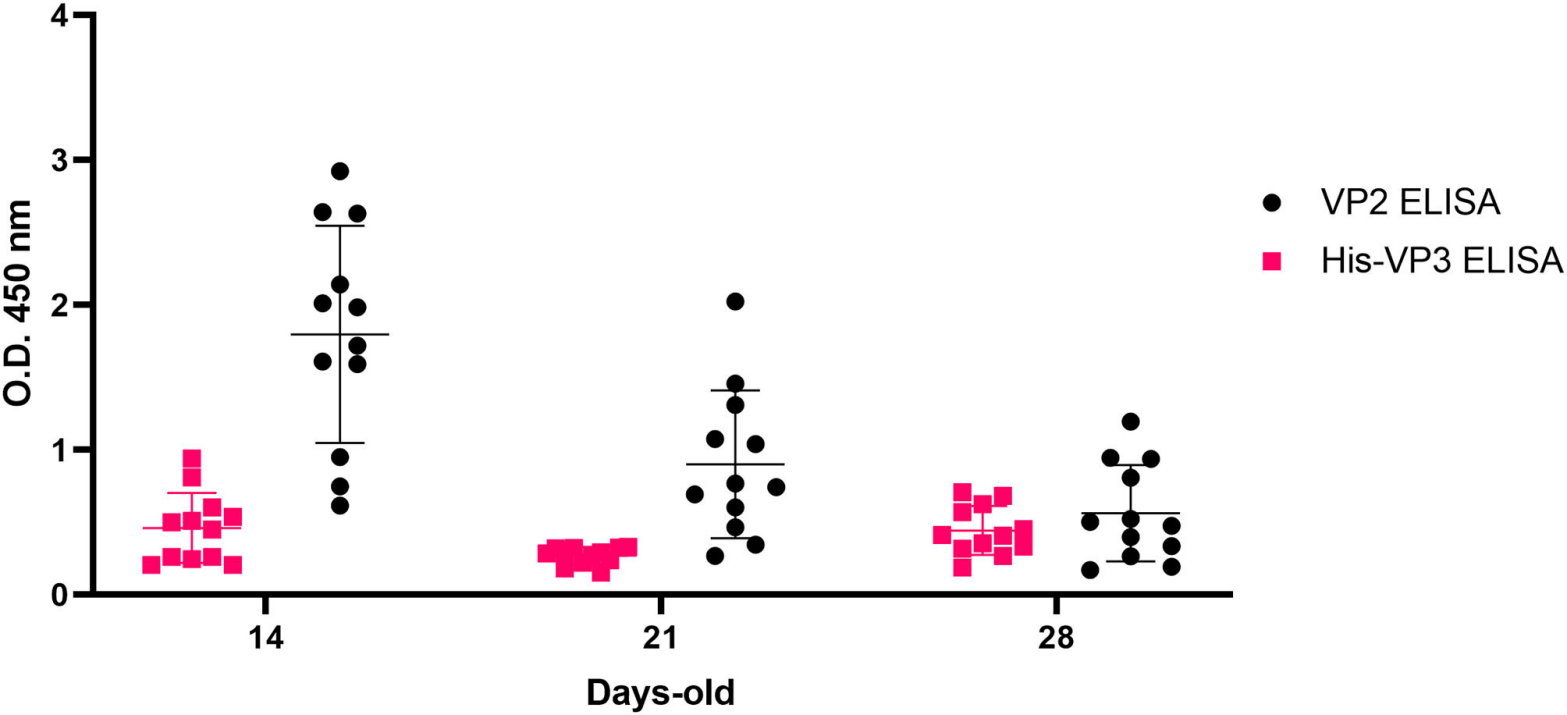
Analysis of VP3-specific MDA in HVT-ND-IBD-vaccinated broiler chickens. Distribution of O.D._450_ values of pre-challenge Broiler-HVT-ND-IBD-vaccinated chickens sera (ID Screen® IBD VP2, black; His-VP3 ELISA, pink), collected at 14, 21, and 28 days of age. Horizontal and vertical bars: mean ± standard deviation (SD).

To this aim, the kinetics of anti-VP2 and anti-VP3 antibodies were studied in the sera collected at 14, 21, and 28 days of age from HVT-ND-IBDV-vaccinated broiler chickens born from inactivated IBDV-vaccinated breeders. This analysis demonstrated the presence of anti-VP3 antibodies with the highest values at 14 days of age (mean O.D._450_ = 0.504), followed by a decrease at Day 21, as observed in VP2-based ELISA. Surprisingly, only in the VP3-based assay, a mild increase in antibody titers was detected at Day 28 (Figure 7).

### Evaluation of His-VP3-Based ELISA Efficacy for DIVA in SPF and Broiler Chickens

To understand the applicability of the His-VP3 ELISA as a DIVA test, a challenge with a field vvIBDV strain was performed in Naïve (control) and VP2-vaccinated chickens (Broiler-HVT-ND-IBD vaccinated; SPF-HVT-ND-IBD vaccinated; SPF-VP2-VLP vaccinated). All the chickens in the control group (SPF-Naïve) showed severe clinical signs (i.e., prostration, ruffled feathers, and difficulty to move) and eight out of 10 succumbed to infection between Days 3 and 4 p.i. (80% mortality). All the birds in the vaccinated groups showed no apparent clinical signs up to 14 days p.i., and no mortality was recorded in these groups.

The rRT-PCR targeting VP4 of IBDV was performed on cloacal swabs collected at Days 4 and 7 p.i. Modest viral shedding (mean Ct = 31.2) in 2/12 (16.7%) Broiler and SPF-HVT-ND-IDV vaccinated chickens were detected only at Day 4 p.i. All the swabs collected from the surviving birds in the SPF-Naïve group (*n* = 2, 100%) resulted positive (mean Ct 28.1) at Days 4 and 7 p.i.

**Figure 8:**
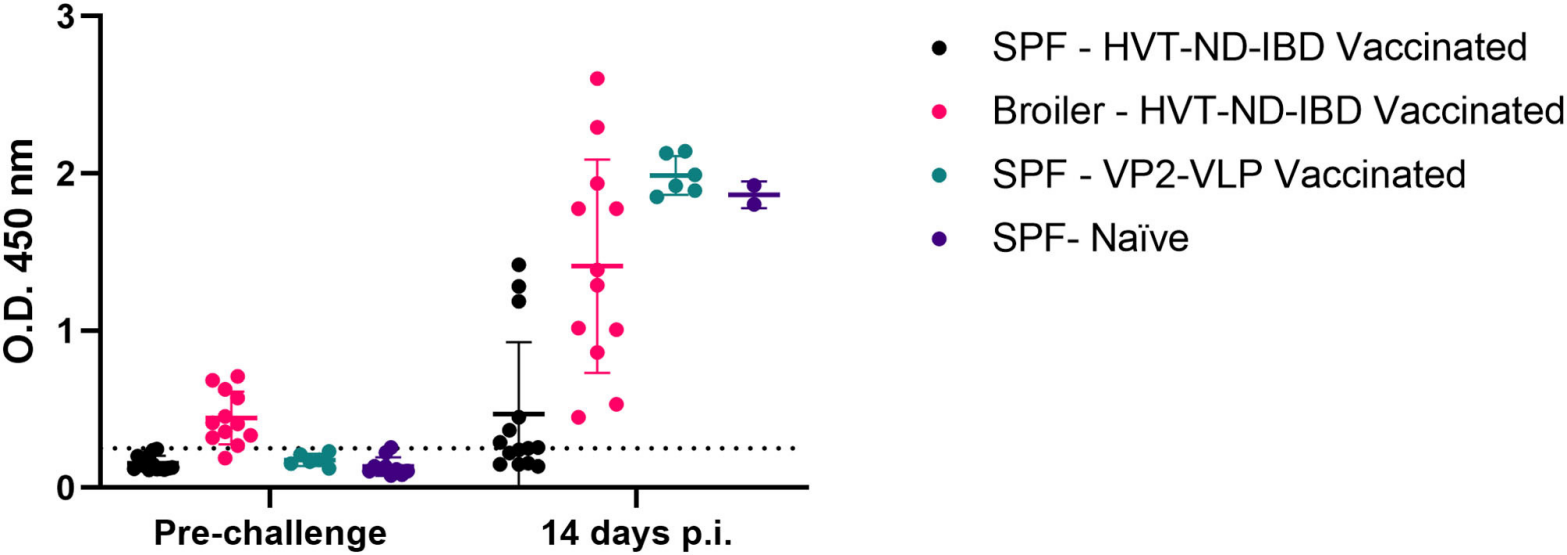
Efficacy of His-VP3-based ELISA in differentiating VP2-vaccinated SPF and broiler chickens before and after challenge. Distribution of O.D._450_ values of SPF and broiler chickens, sera collected before the challenge and after the challenge (14 days p.i.) (SPF-HVT-ND-IBD vaccinated, black; Broiler-HVT-ND-IBD vaccinated, pink; SPF-VP2-VLP vaccinated, green; SPF-Naïve, blue) analyzed with the His-VP3 ELISA. Horizontal and vertical bars: mean ± standard deviation (SD). The positive threshold is indicated with a dotted line (O.D._450_ = 0.250).

The sensitivity of the His-VP3 ELISA to detect infection was confirmed by the analysis of the sera of the chickens immunized with the VP2-VLP vaccine or with the HVT-ND-IBD vaccine before (28 days of age) and after the challenge (14 days p.i.). His-VP3 ELISA values above the threshold were detected in 7/12 SPF-HVT-ND-IBD-vaccinated chickens (58.3%) at Day 14 p.i., while all the vaccinated broilers resulted positive at this time point. Similarly, SPF-Naïve and VP2-VLP-vaccinated chickens were all found positive after the challenge (Figure 8), thus confirming the suitability of the novel ELISA to be applied to detect viral circulation in flocks with different immunological and genetic backgrounds.

## Discussion

Infectious bursal disease control is still one of the biggest challenges for the poultry industry. Over the past 30 years, vvIBDV, which is able to infect and cause disease in chickens, overcoming the barrier represented by MDA against classical strains, has diffused worldwide and caused significant losses (Letzel et al., 2007). Antigenic drift, which for IBDV has been attributed mainly to substitution mutations in the sequence of the hypervariable region of the capsid protein VP2, may result also in a reduction of the efficacy of vaccination (Heine et al., 1991). Viral mutations, reassortment, and recombination have generated also less virulent variant strains and are still able to infect chickens and to produce immunosuppression, and have been found more frequently in subclinically infected animals (Sapats and Ignjatovic, 2000; Lupini et al., 2016; de Wit et al., 2018; El-Aried et al., 2019; Fan et al., 2019). Current IBD control efforts have thus failed to eradicate this infectious disease (Jackwood and Sommer-Wagner, 2011; Müller et al., 2012). Despite the challenges in controlling IBDV circulation, due to the widespread distribution, the high resistance in the environment and the subclinical course of the disease in vaccinated chickens, the development of novel strategies aimed at the eradication of the disease would have a huge impact on poultry production. Recently commercialized live-vectored vaccines and experimental subunit vaccines have offered novel perspectives in disease control, opening the way to the development of novel diagnostic DIVA strategies, leading to disease eradication in well-controlled poultry production systems.

DIVA strategies have been applied with success in the past in the control of avian influenza during the Italian emergency vaccination program between 2000 and 2006 (Capua et al., 2003; Capua and Marangon, 2007) and are also currently applied for the eradication of several important veterinary diseases, such as infectious bovine rhinotracheitis in cattle (Muratore et al., 2017) and Aujeszky's disease in swine (Mettenleiter, 2020).

Multiple commercial IBDV diagnostic kits are available to detect antibodies against the major immunogenic protein VP2, but antigenic drift may reduce the sensitivity of these assays because antibodies raised against mutated versions of the protein are often not cross-reactive (Heine et al., 1991). In this context, VP3 appears to be a very attractive target for the development of diagnostic tools as, in spite of being strongly immunogenic, it is not the target of neutralizing antibodies, therefore not an ideal antigen for the formulation of new-generation recombinant vaccines (Müller et al., 2012). Moreover, the immunogenic epitopes of this protein are strongly conserved among very virulent, classical, attenuated, and serotype 2 IBDV strains (Deng et al., 2007).

Previous publications reported the development of diagnostic assays based on the production of the VP3 protein using *E.coli* (Wang et al., 2008) or insect cells (Martínez-Torrecuadrada et al., 2000) as expression systems. The use of a plant-based expression platform may represent an alternative method for the low-cost production of large amounts of recombinant proteins not only for vaccinal but also for diagnostic use (Rage et al., 2020).

In the present study, the whole VP3 protein sequence derived from a vvIBDV field strain N-terminally fused to a His-tag (His-VP3) was expressed in *N. benthamiana* plants by agroinfiltration and used as a coating antigen to set up a low-cost diagnostic assay with the potential to be applied for DIVA. Western blot analysis of protein extracts obtained from agroinfiltrated tissues revealed that His-VP3 is only partially degraded and specifically recognized by an anti-IBDV chicken serum. The protein was successfully purified from plant extracts using immobilized metal chelate ion chromatography and, when separated on SDS-PAGE, showed also the presence of several high molecular mass bands, possibly indicating the formation of multimeric forms of the protein. This behavior was already reported in plants for antibodies, virus coat proteins, and other soluble molecules such as HIVgp120 and was explained as a possible result from covalent cross-linking of subunits by components, such as peroxidases, in the plant extracts during protein extraction and purification (Castells-Graells and Lomonossoff, 2021). Nevertheless, the coated plant purified His-VP3 was specifically and strongly recognized in ELISA by an anti-IBDV chicken serum.

The His-VP3-based ELISA presented high sensitivity and specificity when tested with a panel of reference sera, including sera derived from animals with immunity toward vvIBDV (naturally infected broilers or experimentally challenged SPF) or from animals immunized with an inactivated IBDV classical strain (D78). High specificity was confirmed also by testing a panel of sera raised in SPF chickens against several important poultry viral pathogens. Similar sensitivity (99 vs. 100%) but lower specificity (89 vs. 94%) has been previously described for an ELISA developed using as coating antigen *E. coli*-expressed VP3 (Wang et al., 2008). The improved performances observed using the plant-expressed product might be related to possible differences in protein folding between the prokaryotic and the eukaryotic system or to the presence of VP3 multimeric forms. The results obtained with the His-VP3 ELISA were perfectly in line (κ = 0.957) with those obtained using a commercial VP2-based ELISA kit, demonstrating that this assay may be usefully applied as a diagnostic tool to identify infected chickens. Moreover, the His-VP3 ELISA was able to detect the seroconversion after the experimental viral challenge of SPF chickens immunized with last-generation VP2-focused vaccines, demonstrating that, when paired with a VP2-based ELISA, it is a suitable DIVA tool. It is interesting to note that, in this study, as well as in previous ones (Marusic et al., 2021; van Hulten et al., 2021), the protection against clinical IBDV induced by both the HVT-ND-IBD and the VP2-VLP vaccine was high as no clinical signs and no mortality were observed (vs. 80% of mortality in the control groups), despite anti-VP2 antibody titers induced by the live-vectored vaccine are lower as compared to those induced by the VLPs. This observation indicates that, in the protection conferred by certain types of vaccines, an important role may be played also by cell-mediated immunity (Dey et al., 2019).

The efficiency of the His-VP3 ELISA was also evaluated in a controlled experimental setting, simulating field conditions, monitoring anti-VP2 and anti-VP3 antibodies in broilers born from breeders vaccinated with inactivated IBDV. The analysis of the sera of these animals, vaccinated at 1 day of age with a commercial VP2-based live vectored vaccine (HVT-ND-IBD), showed that anti-VP2 antibodies titers were high at 14 days of age, consistent with high MDA levels, to then decline. These results are consistent with previous data on immunity conferred by recombinant IBD vaccines in broilers (Le Gros et al., 2009). In the case of anti-VP3 antibody titers, we observed a slight increase at Day 28 and speculate that this might be residual MDA fluctuations or an effect of the influence of the different genetic backgrounds of the birds (Rautenschlein et al., 2007). Nevertheless, the results obtained showed that the introduction of field viruses could be reliably detectable with the His-VP3 ELISA in birds, with MDA starting from 21 days of age.

After the challenge with a vvIBDV, the Broiler-HVT-ND-IBD and SPF-VP2-VLP-vaccinated animals, as well as the two survived SPF-Naïve chickens, were tested positive for anti-VP3 antibodies at 14 days p.i. by His-VP3 ELISA, while only 58.3% of the chickens in the SPF HVT-ND-IBD group were tested positive at the same sampling time. Overall, lower antibody titers against VP3 were detected in both broilers and SPF HVT-ND-IBD-immunized groups after the challenge compared with those detected in SPF-VP2-VLP-vaccinated and SPF-Naïve birds. Similarly, in a previously published study, evaluating IBDV antibody response in broiler chickens immunized with a recombinant HVT-IBD vaccine, the titers after the challenge were low as compared to those of chickens immunized with inactivated, live, or Icx vaccines (Sedeik et al., 2019).

In conclusion, the present study provides the first evidence that the combination of ELISA targeting the VP2 and VP3 proteins could be used to monitor protective immunity and introduction of field IBDV strains in flocks vaccinated with VP2-based vaccines, representing a useful tool for IBDV eradication. Moreover, the His-VP3 protein used in the novel ELISA has been produced in plants, which is a highly scalable and cost-efficient expression system.

## Data Availability Statement

The names of the repository/repositories and accession number(s) can be found below: https://www.ncbi.nlm.nih.gov/genbank/, OK257849.

## Ethics Statement

The animal study was reviewed and approved by IZSVe's Ethics Committee (Authorization N°: n° 709/2020-PR).

## Author Contributions

AB and MD contributed to the conception of the study, performed the experiments, and wrote the manuscript. CM performed experiments and wrote/edited sections of the manuscript. CL edited the manuscript. CD, FG, EM, AF, VP, and FB helped in performing the experiments. SB and CD conceived the study and wrote the manuscript. All the authors contributed to manuscript revision, read, and approved the submitted version.

## Funding

This study was funded by the AVIAMED project through the ERANET ARIMNet2 2015 Call by the following funding agencies: Italian Ministry of Agricultural, Food and Forestry Policies (MIPAAF) and Ministry of Higher Education, Scientific Research and Professional Training of Morocco (MESRSFC). ARIMNet2 (ERA-NET) has received funding from the European Union's Seventh Framework Programme for research, technological development, and demonstration under grant agreement No. 618127/182.

## Conflict of Interest

The authors declare that the research was conducted in the absence of any commercial or financial relationships that could be construed as a potential conflict of interest.

## Publisher's Note

All claims expressed in this article are solely those of the authors and do not necessarily represent those of their affiliated organizations, or those of the publisher, the editors and the reviewers. Any product that may be evaluated in this article, or claim that may be made by its manufacturer, is not guaranteed or endorsed by the publisher.
