# Supplementary material for: Development of a Novel Assay Based on Plant-Produced Infectious Bursal Disease Virus VP3 for the Differentiation of Infected From Vaccinated Animals
Source: Front Plant Sci. 2021 Dec 7;12:786871. doi: 10.3389/fpls.2021.786871 (PMC8689005; doi:10.3389/fpls.2021.786871)
Supplement: Supplementary file 1 [file Presentation_1.PPTX]

## Slide 1
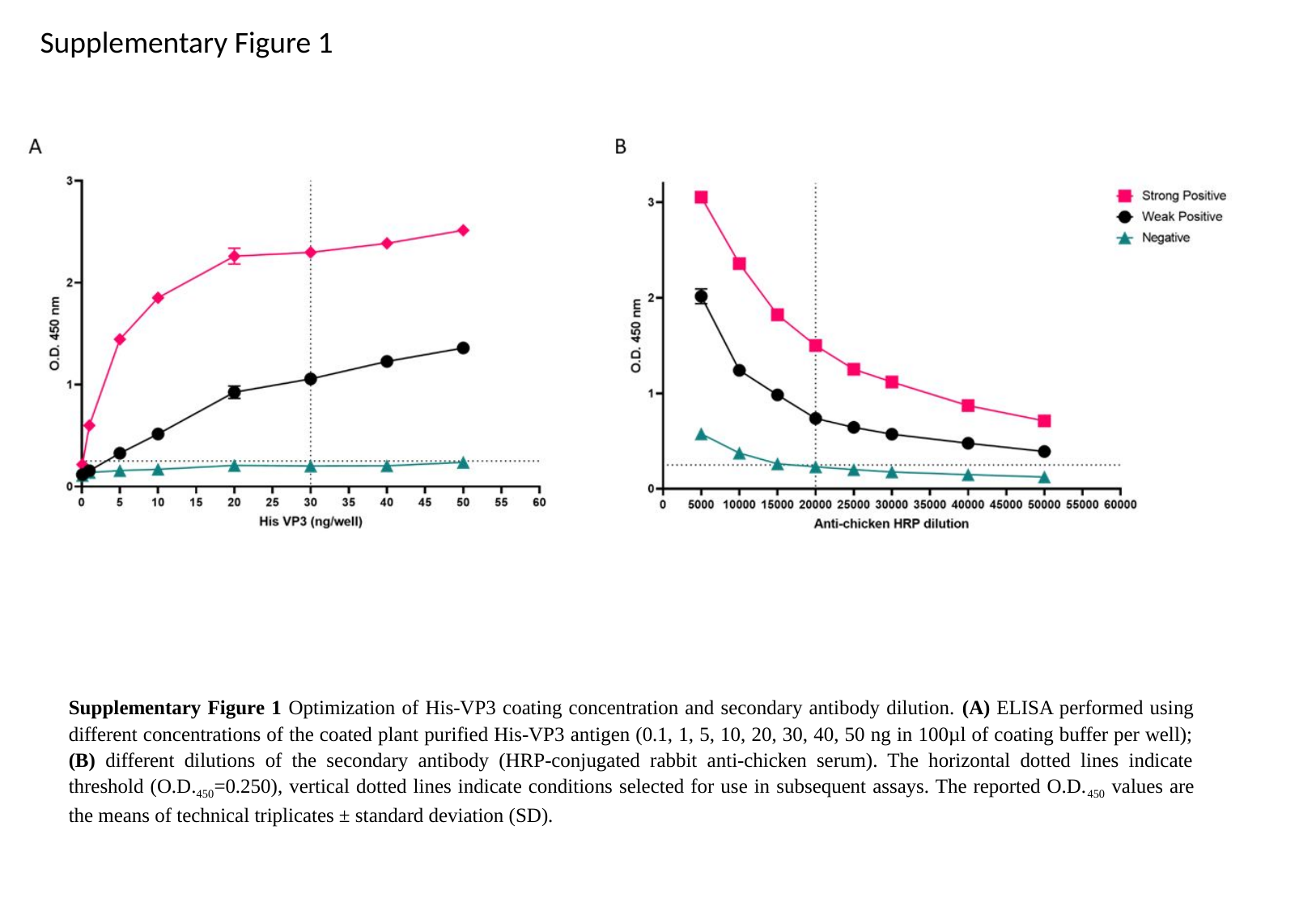

Supplementary Figure 1
Supplementary Figure 1 Optimization of His-VP3 coating concentration and secondary antibody dilution. (A) ELISA performed using different concentrations of the coated plant purified His-VP3 antigen (0.1, 1, 5, 10, 20, 30, 40, 50 ng in 100µl of coating buffer per well); (B) different dilutions of the secondary antibody (HRP-conjugated rabbit anti-chicken serum). The horizontal dotted lines indicate threshold (O.D.450=0.250), vertical dotted lines indicate conditions selected for use in subsequent assays. The reported O.D.450 values are the means of technical triplicates ± standard deviation (SD).

## Slide 2
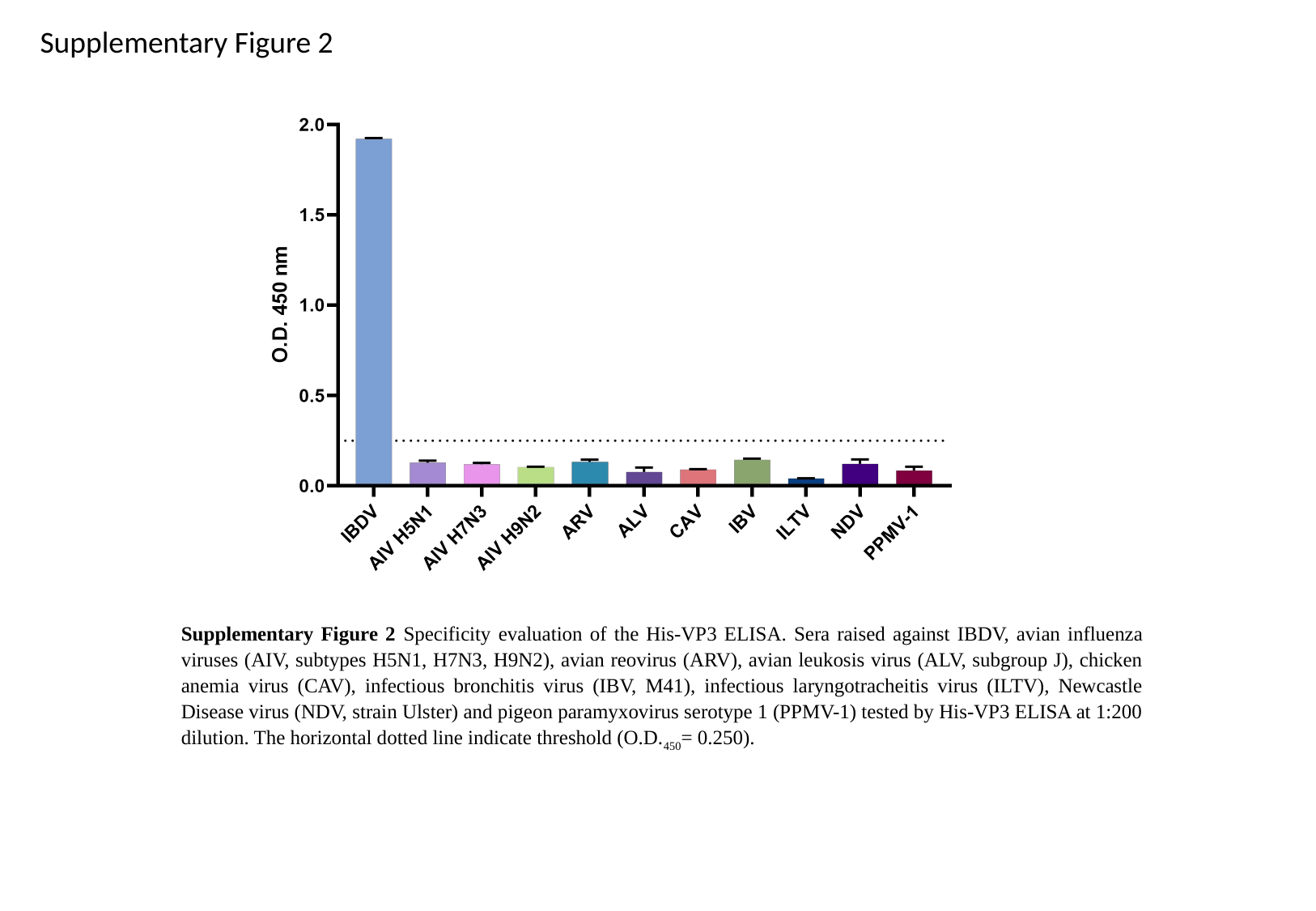

Supplementary Figure 2
Supplementary Figure 2 Specificity evaluation of the His-VP3 ELISA. Sera raised against IBDV, avian influenza viruses (AIV, subtypes H5N1, H7N3, H9N2), avian reovirus (ARV), avian leukosis virus (ALV, subgroup J), chicken anemia virus (CAV), infectious bronchitis virus (IBV, M41), infectious laryngotracheitis virus (ILTV), Newcastle Disease virus (NDV, strain Ulster) and pigeon paramyxovirus serotype 1 (PPMV-1) tested by His-VP3 ELISA at 1:200 dilution. The horizontal dotted line indicate threshold (O.D.450= 0.250).
